# The Osteopath’s Imprint: Osteopathic Medicine Under the Nanoscopic Lens

**DOI:** 10.7759/cureus.33914

**Published:** 2023-01-18

**Authors:** Bruno Bordoni, Allan R Escher

**Affiliations:** 1 Physical Medicine and Rehabilitation, Foundation Don Carlo Gnocchi, Milan, ITA; 2 Anesthesiology/Pain Medicine, H. Lee Moffitt Cancer Center and Research Institute, Tampa, USA

**Keywords:** quantum physics, pain, diaphragm, fascintegrity, myofascial, fascia

## Abstract

Scientific literature demonstrates how osteopathic manipulative treatments (OMT) are able to improve various somatic functional parameters, change somato-visceral and viscero-somatic reflexes toward a more physiological mechano-metabolic environment and, consequently, bring benefits to patients. These benefits can be long-lasting or short-lived. Multiple reasons can be found to explain the positive responses to OMT, ranging from neurological, vascular, lymphatic, and endocrine explanations. Not only the techniques, but the touch of the clinician prove to be important factors for a favorable adaptation by the patient. Another science capable of explaining the change in cellular status and from which reflections that pave the way for observing the human body in a different light can be extrapolated is quantum physics. The latter is rarely taken into consideration to obtain possible explanations of the physical events that occur between the clinician and the patient. The article tries to put the effects of OMT under the light of a new lens: the nanoscopic.

## Introduction and background

Osteopathic manipulative medicine (OMM) through osteopathic manipulative treatments (OMT), demonstrates its effectiveness in numerous clinical and pathological fields. OMT with cardiac surgery patients in median sternotomy, accelerated clinical recovery, with the improvement of respiratory function, pain threshold, and with an early discharge, compared to the group of patients not subjected to the osteopathic practice [1]. OMT improves some respiratory and musculoskeletal function parameters (in the short term) in subjects affected by the chronic obstructive pulmonary disease (COPD), such as the forced expiratory volume (FEV1%), forced vital capacity (FVC), six minutes walking test (6MWT) and COPD Assessment Test (CAT), compared to patients not followed with an osteopathic approach [2]. OMT improves some parameters related to neuromotor coordination in Parkinson's patients, making the complex gait mechanism more stable, compared to patients who did not receive osteopathy [3]. The osteopath with patients suffering from temporomandibular disorder (TMD), is able to reduce the values expressed by an examination with algometry, and improve values inferred from baropodometry and movement of the temporomandibular joint, compared to patients not subjected to osteopathic treatments [4]. Patients suffering from headaches following mild traumatic brain injury may find benefit in osteopathic practice, although the reduction in pain measurable by different tests (Visual Analog Scale, six-item Headache Impact Test) does not persist in a long-term evaluation [5]. Through gentle approaches, OMT is able to bring benefits to patients with non-specific low back pain and with an associated clinical history of urinary tract infections (UTI); improves Kidney Mobility Scores, UTI Symptoms Assessment questionnaire, modified Schober test, and the visual analog scale [6]. There are several reasons given to explain the benefits of the studies cited. The painful and dysfunctional somatic area could be the response of a visceral problem, via a viscero-somatic reflex or facilitated segment [6,7]. By manually working the lumbar myofascial tissue, the afferents that reach the medullary system are improved, in particular, there is a level of conjunction in lamina I of the gray matter of the medulla for somatic and visceral information; somatic fibers and visceral C fibers communicate via interneurons [7]. It is assumed that non-nociceptive afferents reaching the spinal cord are able to improve the response of the autonomic system (sympathetic and parasympathetic) which will positively influence the alpha and gamma neurons of the musculoskeletal innervation segment and the afferent/viscera-related efference (myotoma and viscerotoma, respectively) [7]. A somato-visceral circuit is created, improving the viscero-somatic response. The synaptic relationships, enriched by new non-dysfunctional information, will improve the innervation areas of other neighboring tissues, positively involving the specific sclerotomes, angiotomes, and dermatomes [6]. Patients with headaches following mild traumatic brain injury may have benefited from the creation of a more favorable mechano-metabolic environment [5]. Improving the physiological articular movement of the cervical zygapophysial joints could have raised the activation threshold of local nociceptors, decreasing afferential hyperexcitation [5]. Afferent fibers from C2-C3 roots anastomose with the spinal trigeminal nucleus, which sends information to Gasser's ganglion; this link could be one of the causes of pain or benefit experienced by patients undergoing OMT [5]. The sub-occipital muscle area is in direct contact with the dura mater, via myoconnective tissue bridges or myodural bridges; in addition to the high percentage of mechanical receptors, these muscles facilitate the flow-outflow of cranial fluids (cerebrospinal fluid and glympha) [8]. Probably, gentle manual approaches here could have facilitated the flow of fluids, decreasing any non-physiological pressures. Decreased blood pressure may have decreased activation of dural trigeminal receptors. Patients with TMD and approached with OMT, have made some improvements. The pain threshold was elevated, increasing the joint range of motion [4]. One of the reasons given by the authors is a decreased local sensitivity to mechanical stimuli. The temporomandibular joint (TMJ) and related structures (muscles, capsule, retrodiscal area) are innervated by the mandibular nerve and some of its branches (posterior deep temporal nerve, masseteric nerve, auriculotemporal nerve) [9]. The trigeminovascular system can be a source of pain, involving different areas of the face, including the TMJ area, with increased central and/or peripheral sensitization [10]. The calcitonin gene-related peptide (CGRP) can be synthesized paracrinely by trigeminal neurons; the increase of this neuropeptide stimulates an inflammatory environment and increased nociceptive afferents [10]. OMT could improve the movement complex of the TMJ and, probably, stimulate the secretion of substances able to decrease the production of CGRP, increasing the GABAergic system [11]. OMT administered to patients with Parkinson's improves some neuromotor parameters; an explanation highlighted by the authors is that of having improved some cranial dysfunctions [3]. Probably, by manually working the skull tissues, the surface receptors of the trigeminal system are stimulated, which can send afferents to the cerebellar and vestibular area, positively influencing postural balance [12,13]. The osteopathic approach for patients with COPD, combined with rehabilitation and pharmacological treatments, could demonstrate a greater benefit for this clinical setting. In the literature, there is no univocity in stating that OMT is absolutely valid for respiratory patients [14]. The multiple explanations of the positive results obtained are always linked to structural factors, such as the improvement of the movement of the ribcage (joints and muscles) [14]. Osteopathy in cardiac surgery patients, undergoing median sternotomy, has highlighted several benefits, including an increase in the pain threshold [1,15]. A possible reason for the reduction of nociceptive phenomena recorded by the patient could be linked to the stimulation of the parasympathetic system, compared to a more active sympathetic system after surgery [16]. A gentle manual approach is able to stimulate the parasympathetic system; the latter is linked to greater pain control, probably due to the nociceptive relations reaching the nucleus tractus solitarius (NTS) and the management from the latter toward other brain areas (anterior limbic area) [15,17]. Another hypothesis is that of the activation of the central descending pain inhibition pathways [17]. The article aims to try to understand the positive effects resulting from the OMT approach under the lens of the vision of physics, trying to have a point of view from the microscopic to the nanoscopic.

## Review

Microscopic mechanisms affecting visible matter: fluids

**Figure 1:**
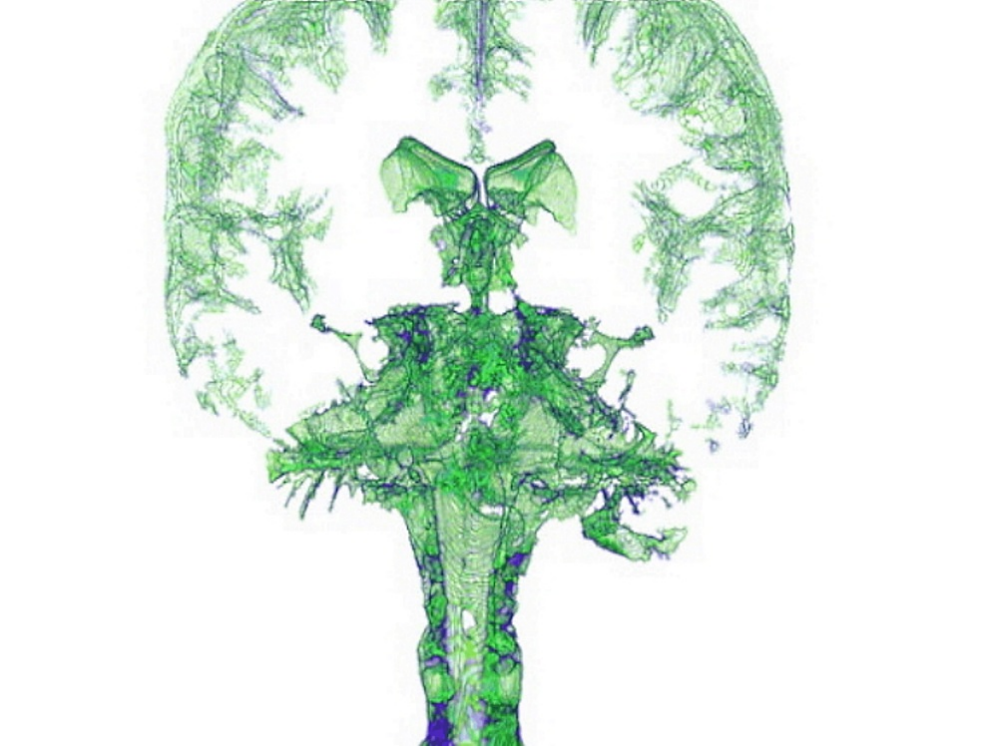
The image illustrates the distribution of the brain and part of the medulla of the cerebrospinal fluid (green color). Thanks to the introduction of new software and new magnetic resonance machines (3 Tesla), it is possible to represent the distribution of the cerebrospinal fluid. Image by Bordoni Bruno.

Fluids, such as blood, lymph, and cerebrospinal fluid, determine bodily form and function [18]. Fluids carry not only biochemical information but electrical and magnetic (electromagnetic waves) information throughout the body [19]. For readers who wish to deepen this last assertion, we recommend the article by Savelev and Myakishev-Rempel (Evidence for DNA resonance signaling via longitudinal hydrogen bonds. Prog Biophys Mol Biol. 2020 Oct;156:14-9). The viscosity of the fluids influences the entity of the electromagnetic oscillations of all the tissues with which they interact, while the elasticity of the same fluids will influence the frequency of the oscillations. Unlike electromagnetic waves in a vacuum, which are linear and can therefore pass through each other unchanged, the waves of fluids in a nonlinear medium interact with each other; fluids affect other fluids. Fluids possess different types of molecules (isotropic and anisotropic), nematic-chiral structures (non-constant molecular orientation), nematic structures (there is a preferential orientation), and smectic structures (preferential orientation and ordered with less free movement) [20]. The body fluids themselves can be defined as mesogenic structures, molecular complexes with different orientations and degrees of movement (mesophases) [21]. These molecules and cells change their position depending on the electric or electromagnetic field they come into contact with (superficial or deep), or the magnitude and electromagnetic frequency they carry. For example, the simultaneous presence of several non-homogeneous vortical structures (cells and molecules in the blood), which mutually interact with each other (non-linear structure of the Navier-Stokes equations), can change the electromagnetic information carried, influencing the viscosity of the blood. If blood viscosity increases, the electromagnetic wave vectors (axial and azimuthal) can become opposing forces that collide or create friction [22]. This, in the long run, could lead to pathologies (Figure 1).

We can imagine fluids as a stratified network, as they possess different elasticity and energy within which a second network of vital information simultaneously resides. At the cellular level, fluids are capable of influencing the size of the cell surface, and the greater this change over time (the more mechanical stresses and the more morphological changes), the greater the aging of the cell itself. In fluid dynamics, a turbulent regime is a motion governed by the laws of chaos; chaos or entropy allows the maximum possible adaptation and maximum efficiency [23]. The absence of entropy or negentropy will determine the presence of patterns, that is, a structure (large or small) will be bound in a predetermined space or direction, and this situation of non-total freedom will lead to pathology [24]. We could imagine fluids as the “soul” of solid bodily matter. Each tissue is immersed, enveloped, and penetrated by fluids. For example, the volume of skeletal muscle is given by about 80% of the presence of fluids; the movement of these fluids allows the muscle tissue to adapt and communicate (within the muscle and with neighboring muscles) [25]. The same force produced by the muscle is given, in part, by the presence of increased internal pressures during contraction, reducing the effort deriving from the biochemical production of energy. This allows the smaller muscles to generate sufficient force to perform their function, and to create a fluidic pressure network that connects other muscles (spinal muscles), producing a functional system [25]. Every time we put our hands on the patient, we are acting on the fluids and, possibly, on the whole body.

Microscopic mechanisms affecting visible matter: microRNAs and nanotunnels

Looking further into the human body through a microscopic lens, we discover that there are different strategies used by cells and cellular components to communicate over great distances. microRNAs (miRNAs) are derived from each cell and travel throughout the body through fluids; they are small molecules, with a maximum of 21-23 nucleotides. They are non-coding RNA (RiboNucleic Acid) molecules capable of influencing various cellular processes, such as apoptosis, proliferation, differentiation, migration, and metabolism [26,27]. A single miRNA can influence hundreds of different RNAs, and conversely, a single RNA can be controlled by different miRNAs [27]. miRNAs play essential roles in gene expression. There is no research that has evaluated the plasma presence of miRNAs after an OMT approach. Recently, an increase in circulating miRNAs is synonymous with the presence of various pathologies and neuropathic disorders, and they are used as new clinical markers [28]. Considering that OMT improves the nociceptive aspect, we could hypothesize that a decline in the plasma values of these nucleotides occurs, although there is no study that evaluates the circulating quantity of miRNAs in the osteopathic field. miRNAs can travel together with other biochemical structures, through mobile vesicles, which always allow a long-distance dialogue between different tissues and cells. These extracellular vesicles (exosomes, endosomes, and ectosomes) carry numerous signals and information, which influence the function and metabolic environment of their target [29,30]. Each cell is able to form nanotunnels to communicate with nearby and distant cells, and/or form such tunnels for molecular exchanges between multiple cellular structures of the same cell. Mitochondria are capable of forming nanotunnels and influencing not only the behavior of the neighboring mitochondrion but the entire cellular behavior [31]. A single mitochondrion can contact one or more mitochondrion and eventually fuse and form a new mitochondrion [31]. Telocytes (interstitial or stromal cells), are present throughout the body, can communicate through long “tentacles” that reach between tens and hundreds of micrometers, called telopods [32]. Telopods touch not only other telocytes but cells of different nature, close and distant [33]. Nanotunnels allow to transport different biochemicals and extracellular vesicles for the regulation of cellular morphogenesis, regeneration and metabolism, and influence the function toward a physiological or pathological environment [34]. Another cell that is always present in the body is the fibroblast. The latter communicates with other fibroblasts and different cells through nanoscopic ramifications or dendritic extensions (less long than telocytes), contributing to the function of the tissue in which the same fibroblast lives [35]. We know that the application of manual therapies influences the spatial orientation of fibroblasts and their tissue behavior [36]. Currently, we do not know whether OMT is able to change the form of communication between cells, by stimulating additional nanotunnels.

Nanoscopic mechanisms affecting visible matter: holographic fascia

**Figure 2:**
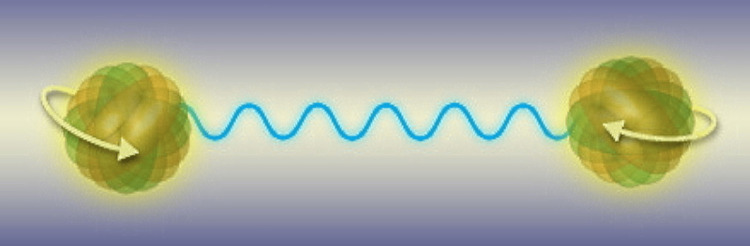
The figure schematically represents two biophotons and the theme of the image is to remind that when two cells, two electrons and other living structures come into contact, this contact will be forever (in space-time), thanks to the phenomenon of entanglement. Image by Bruno Bordoni.

The elementary particles that makeup matter as we know it are the boson and the fermion. The behavior of these particles can be measured as a wave function (oscillation) as they exchange electrons. We can think of the wave function as a limit vector of infinite and continuous components; in quantum mechanics, the concept of the trajectory of classical mechanics does not exist, which presupposes the simultaneous knowledge of the position and velocity of the particles. Every particle has a spin. The spin (intrinsic angular momentum) of an electron, in quantum mechanics, is a quantity describing a classical mechanical angular momentum based on the rotational motion of a mass; in quantum mechanics spin is a quantity, or quantum number, associated with particles, which contributes to defining their quantum state [37]. The fermion, like electrons, protons, and neutrons, is responsible for solid matter; a fermion has an odd or antisymmetric structure in its orbit [38]. Bosons (such as photons - biophotons and phonons - biophonons) have equal and symmetrical structures on their orbits and constitute the different forces that allow exchanges between atoms (electromagnetic forces, light, sound) [38,39]. We are the macroscopic expression of the coherence of structures conceivable from a nanoscopic vision. The concept of nanoscopic vision can be used to understand the human body and its smallest reactions and functions [40]. For example, neutrophils are among the first cells that rush in case of inflammation, with micromicidal functions. The microbicidal action takes place through a combustion, moving some electrons from the most peripheral orbits of the oxygen molecule, transforming a fermonic structure into a bosonic [37]. This combustion produces light (bioluminescence or chemiluminescence), that is, particles of light or photons (biophotons) [37]. Oxidoreductase in biochemistry is the transfer of electrons between molecules. This transfer could use the phenomenon of quantum tunneling, that is, moving electrons on the orbits of different molecules without necessarily using chemical energy [40]. When the oscillation or vibrational frequency of one molecule is recognized by another molecule (a quantum receptor form), electrons can be transferred creating an electrical signal [40]. A quantum state (vibrational identity) affects the electrical state and ultimately the deformation of the cell (mechanotransduction). All living cells are able to communicate at the quantum level [41]. Another phenomenon that explains the behavior of the cell is entanglement. Through the phenomenon of entanglement, the electrons that come into contact will produce the same behavior (close or distant), establishing an indestructible bond (Figure 2).

Deoxyribonucleic acid (DNA) is a protein double-stranded oscillatory structure; the oscillations move the electrons and change the behavior of the DNA [19]. Quantum tunneling changes the oscillation of DNA helices, imprinting mutations (physiological and non-physiological), influencing protein folding and polymerase movement [40,42]. Complex living systems (man) have a bi-directional (entanglement) and constant quantum relationship with the environment in which he lives (epigenetics) [42]. Fermions and bosons collect and exchange information (senes) at the quantum level; the information will then be managed and encoded by the DNA (senoma). The senoma or processed information or consciousness travels faster than the classic electrical or chemical exchange between cells and, once in contact with other particles, every cell and every macroscopic living system embodies the genetic awareness of the people and the environment in which lives [19,43]. With the vision of quantum physics in biology, there is no question of distance or time. Looking at the body with quantum optics, our material boundaries vanish, the body does not end with the epidermis, but continues and evolves through electromagnetic oscillatory information (biophotons and biophonons), which the body emanates and receives; as written in our previous work, we can speak of a holographic fascia [19].

Quantum physics and the osteopathic touch

The perception of reality does not depend on the observed reality, but on the observer: there is no objectivity, but subjectivity [44,45]. The observer is able to influence the behavior of the electrons (intention), which electrons involved in the entanglement, allow us to understand the behavior of distant electrons, observing the closest electron [44,45]. These concepts are part of the “observer theory” of quantum physics [45]. Bringing these concepts back to the practice of osteopathic medicine, we could hypothesize that not only is the operator able to deeply perceive the patient's body system, but the same patient simultaneously perceives the osteopath's body system. Every osteopathic manual evaluation is a mutual exchange of information through nanoscopic forces. The cellular DNA of the osteopath and the patient, according to quantum principles, share the same epigenetic memory, and will always be connected, influencing each other. Osteopathic touch is not just knowledge through palpation, but an act of mutual sharing. Unlike the mechanistic view, quantum physics has developed other concepts about form and function as usually perceived by the senses. Form is memory, while function is communication [46]. The aware cell (capable of changing behavior), despite the lack of boundaries at the nanometer level, allows the maintenance of memory through communication. The resulting awareness is not a mere reflection, as it allows the persistence of such an equilibrium for which continuity is implemented. The fascial continuum is the macroscopic expression of this memory. If the memory becomes ineffective (impaired communication), the disease appears. We can ask a question: can the ineffectiveness of decreasing the expression of the disease by the osteopath derive from a lack of effective communication between operator and patient? We do not know. We know that every act is unrepeatable and unmeasurable: subjectivity is not comparable [47]. Probably, the osteopath's touch will be effective if the vibrations of the molecules coincide or tune into the patient's vibrations, leading to a change in more physiological cellular behavior. What you touch stays in your memory. What touches us remains in memory. How we touch comes from the integration of memory. How we feel what touches us comes from memory processing (physical and emotional memory). The imprint of the touch remains on the patient, just as the operator retains what he feels. Osteopathy is not an act. It is a constant flow of interpenetration and knowledge: it is the encounter of man with man, sublimating wholeness in a gesture. The subtle forces that bind multiple molecules of matter as we know it are the missing key to understanding health and disease [48]. There is no neutrality, but balance (in health); there is no immobility, but constant motions of different electromagnetic spectrums. Silence does not even exist, because every exchange of electrons produces sound (phonons) [19]. The hand should not seek neutrality or silence; the hand should be looking for functional entropy. The latter is the maximum adaptability of the organism which is expressed in health [48,49]. Conversely, the presence of dysfunctional syntropy implies the presence of a predominant pattern that disrupts the balance and leads to disease [48,49]. The incessant exchange of information at the sub-atomic level allows for the maintenance of human form and function and health [50-52]. The glue that holds cells together is electromagnetic forces (biophotons and biophonons) [52]. Body fluids are essential for the optimal transport of electrons (negative charge) and protons (positive charge), biophotons and biophonons affecting bodily health, starting from the nanoscopic [52]. The change in the electrical spectrum generated by the passage of fluids generates a magnetic field (electromagnetic field), which will become an oscillation of biophotons and biophones. Electromagnetic fields millions of times weaker than the electrical voltage of the cell membrane are capable of manipulating the metabolic behavior of the human body [52]. How can we put this information into action? We cannot manually know the level of oscillation that determines cellular health; however, we can put the intention and tune into the flows of body fluids, searching with palpation for the maximum movement allowed by the patient's body through fluidic movements. Since everything in the body is inseparable, everything is attainable. It is not the technique that affects the salutogenic stimulus, but the awareness of the operator. And since there is no distance, there is no need to move, that is, it is not so much necessary to use invasive techniques (for example, the high-velocity low-amplitude-thrust approach). The complexity is within the infinitesimal, beyond which there is the simple. The smallest action reverberates deep inside the whole. Osteopathy is non-action towards entropic balance.

## Conclusions

Subtle electromagnetic forces govern the human body, and the same forces of the environment around us are able to influence us. With a nanoscopic vision linked to quantum physics, our body is an infinite network of constant exchanges of energy, in which energy in the form of bosons and fermions determines form and function. Osteopathic touch, as an integral part of this electromagnetic network, and the patient, form a profound interconnection. The osteopath could affect at a quantum level through very delicate manual approaches, focusing his attention on the motion of body fluids. The scientific community should make a further effort to observe the human body from the nanoscopic lens.
